# Outcomes of management practices on regenerative and organic cattle and sheep farms in the UK—a systematic literature review

**DOI:** 10.1007/s13165-026-00559-3

**Published:** 2026-05-09

**Authors:** Laura Freeland, Louise Whatford, Christina L. Marley, Christopher J. Sandom, Barbara Häsler

**Affiliations:** Veterinary Epidemiology, Economics and Public Health Group, Department of Pathobiology and Population Sciences, Royal Veterinary College, London, UK; Veterinary Epidemiology, Economics and Public Health Group, Department of Pathobiology and Population Sciences, Royal Veterinary College, London, UK; Animal Welfare Science and Ethics Group, Department of Pathobiology and Population Sciences, Royal Veterinary College, London, UK; Institute of Biological, Environmental and Rural Sciences, Aberystwyth University, Aberystwyth, UK; School of Life Sciences, University of Sussex, Sussex, UK; Veterinary Epidemiology, Economics and Public Health Group, Department of Pathobiology and Population Sciences, Royal Veterinary College, London, UK

**Keywords:** Regenerative agriculture, Organic, Farm management practices, Livestock

## Abstract

Livestock farmers in the United Kingdom must balance productivity and economic viability with numerous sustainability goals, particularly those related to achieving net zero and nature restoration. Resultantly, interest is growing among stakeholders in organic and regenerative agriculture as potential routes to a sustainable food future. To explore the sustainability of these systems, a systematic literature review, following the PRISMA protocol, was conducted comparing the management practices used on organic and regenerative cattle and sheep farms in the UK. The relationship between farming system and management practices on outcomes for animal health, animal welfare, productivity, finances and bio-diversity were compared to conventional. A total of 45 papers were included, revealing a gap in research on regenerative ruminant systems (2 documents), compared with organic systems (43 documents). Research on organic farming demonstrates that, compared to conventional farming, financial performance is improved, productivity is decreased, and no difference is frequently observed on animal health and welfare, and biodiversity. In contrast, empirical research on regenerative livestock farms only assessed earth-worm abundance, as a soil health indicator, indicating the need for studies examining a broader range of outcomes. Comparisons were not possible between farm types due to heterogeneous study designs, highlighting the need for standardised methodologies to produce comparable evidence on sustainable farming practices. Methods used in organic research may be valuable for the design of studies evaluating regenerative livestock systems. Key management practices undertaken on livestock farms included rotational grazing, use of multi-species pastures, crop-livestock integration and improving biodiversity via practices like hedgerow maintenance.

## Introduction

Farmers in the United Kingdom (UK) face increasing demands as modern food production must align with multiple sustainability goals including biodiversity conservation, climate change mitigation (United Nations 2023), and increasing expectations of high animal health and welfare standards (Government Food Strategy 2022), while under growing economic uncertainty (Uberoi 2023). This challenge is particularly pronounced for meat sheep and cattle farming, which occupies approximately 45% of agricultural land for grazing and mixed arable-grazing systems (Defra Farming Evidence Pack 2024), in addition to the land used to grow approximately 5.7 million tonnes of animal feed (Defra Chapter 9: Intermediate Consumption 2024). With a population of 9.3 million cattle and 30.5 million sheep (Livestock Populations 2025), the industry generates approximately £6.3 billion in dairy products, £4.1 billion in beef and veal, and £1.8 billion in mutton and lamb (Accredited Official Statistics Summary 2025). It is estimated that 97% of farms are considered conventional in the UK, operating outside of organic certification (DEFRA Farming Evidence 2025). While this term encompasses a wide range of systems, it is typically associated with use of external inputs such as concentrate feed and fertiliser to maximise production.

To better balance food production and sustainability needs, interest is growing among farmers, governments and research communities in alternative farming approaches such as regenerative and organic agriculture. Organic livestock currently only accounts for a small proportion of total ruminant numbers, with around 2.9% of cattle and 2.1% of sheep reared under organic certification in 2024 (Defra Organic Farming Statistics 2024). The proportion of the industry under regenerative management is currently unknown, however 121,000 acres of land have been self-declared by farmers as regenerative, equating to 0.29% of all farmland in the UK (RFOU 2026). By comparison, 3.3% of land is declared as organic (Defra Organic Farming Statistics 2024). Given the current scale of alternative approaches, shifts towards these systems could have significant impacts making it important to understand the outcomes of such systems. Therefore, the way the land is farmed will play an important role in achieving the UK’s nature recovery and net zero ambitions, with choice of management practices and farming system significantly impacting sustainability outcomes.

Findings from Newton et al. (2020) suggest regenerative farms have the goal of improving, or regenerating, the natural environment through holistic thinking and a focus on soil health. A focus on soil health is considered central to regenerative agriculture, as demonstrated by regenerative principles set out by influential organisations such as Groundswell (Ritz 2021). However proponents of regenerative agriculture also emphasise the need for financial viability of farms and context-specific management practices to maximise benefits (Tittonell et al. 2022). The absence of agreed standards or definition has resulted in a situation where regenerative agriculture may be defined differently by organisations and individuals depending on their own interests. This contrasts with organic agriculture which is strictly defined in the UK due to long-established certification systems. Organic standards require farms to comply with last-resort use of antibiotics, higher animal welfare standards than the legal requirement, including continuous access to pasture when weather and ground conditions allow, and prohibition of genetically modified organisms and synthetic chemicals (e.g. Soil Association Standards 2024). The lack of differentiation between systems complicates research that aims to establish the outcomes of different farming systems to inform decision making.

Identifying the characteristics of different farming systems and the management practices undertaken on them can help to differentiate systems and document their outcomes. This is particularly important for unregulated approaches such as regenerative, to avoid potential greenwashing by companies selling ambiguously labelled produce. Newton et al. (2020) recognised that farming systems can be described as a set of practices or intended outcomes, but the latter provides a challenge in identifying farms as outcomes may not have been achieved. In this systematic literature review (SLR), a management practice is defined as a method or action relating to raising livestock or managing the farm. Identifying the management practices used within different farming systems provides farmers with a clear strategy and enables researchers to differentiate one system from another. This is especially important in regenerative agriculture, which is gaining interest (Tittonell et al. 2022) but has been characterised in a range of ways and little researched (Newton et al. 2020). A drawback of identifying farms based on management practices is that a lack of set requirements is seen as a benefit by many, allowing for creativity, experimentation and context-specific practices. Hence an in-depth understanding of the management practices used between different farming systems may allow for key differences to be identified for research whilst still allowing flexibility in the choices made on farm.

Determining how farming systems and management practices perform with respect to sustainability outcomes in the UK is highly relevant, particularly in the wake of post-Brexit agricultural transitions taking place between 2021 and 2027 (Defra Agricultural Transition Plan 2020). By 2027, payments to farmers will no longer be tied to land ownership, but to actions that are seen to lead to improvements in animal health and welfare, the environment, and reductions in carbon emissions. Farmers will be paid for actions such as nature and landscape recovery, or provided with grants to purchase equipment to reduce stress in livestock. At the time of writing, the main agri-environment scheme in England, the sustainable farming incentive (SFI), is closed to new applications after funding for the current round was fully allocated in March 2025. The scheme is undergoing reform and expected to reopen for applications in June 2026, with the aim of having fewer actions, reduced complexity and a payment cap (SFI26 2026). Overall, these policy shifts present both opportunities and challenges for farmers as they adapt to new sustainability expectations and financial support.

Developing a comprehensive understanding of the management practices and outcomes of agricultural systems in the UK setting is crucial to guide policy, inform farmers’ decision making and shape business strategy. This is particularly important for regenerative agriculture as numerous major pledges and road-maps for advancing sustainability now incorporate regenerative practices (McCain 2024; Nestle 2024; Defra 2020; Unilever 2020). It is also essential to consider the outcomes of farms holistically, not in isolation, to build a picture of how these outcomes interact within the farming landscape.

To our knowledge, no reviews have been conducted to date comparing the management practices and outcomes of organic and regenerative farming in the context of UK ruminant livestock farms. These two farming systems were chosen as the dominant alternative ruminant farming systems in the UK. The aim of this systematic literature review (SLR) was to compare the management practices used on organic and regenerative cattle and sheep meat farms in the UK and describe the relationship of farming systems and practices with outcomes of animal health, animal welfare, productivity, finances and biodiversity. These outcomes were chosen as they are key indicators of farm sustainability, and relevant to current priorities in the UK. The objectives were to 1) identify the management practices used on organic and regenerative farms, 2) determine whether farming systems or practices affect animal health, animal welfare, biodiversity, finances and productivity, 3) identify the methods used to assess these outcomes, and 4) compare practices (by identifying similarities and differences between farming systems) and outcomes (by summarising findings and reporting significance levels) of each farming system to one another and to conventional systems.

## Methods

### Search strategy and criteria

PRISMA guidelines were followed in undertaking and reporting this review (Page et al. 2021). Trial searches of titles and abstracts were performed within seven databases to determine the most effective search terms and databases to use: Web of Science, Wiley Online Library, PubMed, Scopus, Science direct, JSTOR and Google Scholar. Boolean operators were used to combine terms and phrases to create the final search term (Table 1).

PubMed did not allow for wildcards with less than four characters, so all variations of shorter terms were entered. JSTOR and ScienceDirect did not permit enough characters or Boolean connectors per search and were therefore excluded. The searches in Web of Science, Scopus, Wiley Online Library and PubMed were completed on 28/03/2024.

As Google Scholar searches returned 259,000 results using the search term, the first 150 papers (15 pages) were exported into Excel (Microsoft Corporation 2025) and screened for the full set or stopped earlier if 3 consecutive pages with no relevant results were returned. A threshold of 150 papers was chosen as historically, studies found 100 to be sufficient (Hughes et al. 2014; Reed et al. 2015; Roe et al. 2014), but other authors suggested increasing this number to capture further grey literature (Haddaway et al. 2015). Google Scholar searches were completed on a later date than initial searches (01/05/2024) as the list of documents could not be automatically exported; each source had to be manually extracted into Microsoft Excel.

### Selection process and data extraction

Downloaded search results from Web of Science, Scopus, Wiley Online Library and PubMed were imported into Rayyan (Ouzzani et al. 2016), where duplicates were removed using the duplicate removal function. Title and abstract screening were conducted by L.F. and L.W. using Rayyan’s blinded system, and by using the hide row function in Excel for the Google Scholar papers. Papers were included if they reported on cattle and/or meat sheep in the UK, kept in organic or regenerative systems, and addressing at least one of the following outcomes: animal health, animal welfare, biodiversity, finances or productivity. This review included social and natural science papers. Social science papers were eligible where they examined stakeholder perceptions or reported experiences relating to these outcomes. Including both measured outcomes and stakeholder perspectives was considered important in understanding farming systems. Papers on dairy sheep production were excluded as this practice is not commonplace in the UK. Any documents where it was unclear if the inclusion criteria were met from the title and abstract were classed as a ‘maybe’ and subjected to full-text evaluation, for example, if the location was unknown. Rater agreement was 90% for the papers in Rayyan and 81% for the papers in Excel from Google Scholar. Papers for which there was disagreement between raters were discussed until an agreement was reached.

All documents that met the inclusion criteria, or those that were considered as a ‘maybe’ via the title and abstract screening were read in full. Documents were then excluded if they were identified as commentaries, poster abstracts, reviews with no relevant references, older versions of documents that were already included, papers where it was not possible to separate relevant and irrelevant data (e.g. studies reporting combined outcomes for multiple species) and conference proceedings. Included documents were either peer-reviewed publications, reports and case studies with reproducible methods, or reviews. Relevant review papers were included in the total number of documents, however meta-data was not included in analysis. Full books were assessed using the titles and abstracts of relevant chapters using the same criteria as described above. The reference lists of each document included were checked for potentially relevant publications missed in the original search and subjected to title and abstract screening and subsequent steps as described above.

All documents included in the final list had the following information extracted into Microsoft Excel: information on title, first author, date of publication, farm type, target population, location, outcome, summary, management practices mentioned, methods, data type (primary, secondary or review), simple description of results and outcomes, and whether the data was evidence or opinion.

### Data analysis

Management practices were grouped by farming system and a count of the number of sources mentioning a practice for each species was tabulated. The percentage of publications referencing each practice within each farming system was calculated and presented using a heatmap. Studies were grouped into four categories; namely studies that i) compared outcomes of a farming system to conventional farms, ii) undertook comparisons on a single farm type, iii) reported on outcomes with no comparison, and iv) presented opinions.

For each category, the farm type and outcome were presented as simple counts and proportions. Methods for all studies were counted and grouped by outcome assessed. The number of publications on each farming system, over time, was presented as a bar chart, and the focal species of each study were presented as a Venn diagram. Data presentation was undertaken in Microsoft Excel.

## Results

### Search and Screening Outcomes

**Fig. 1:**
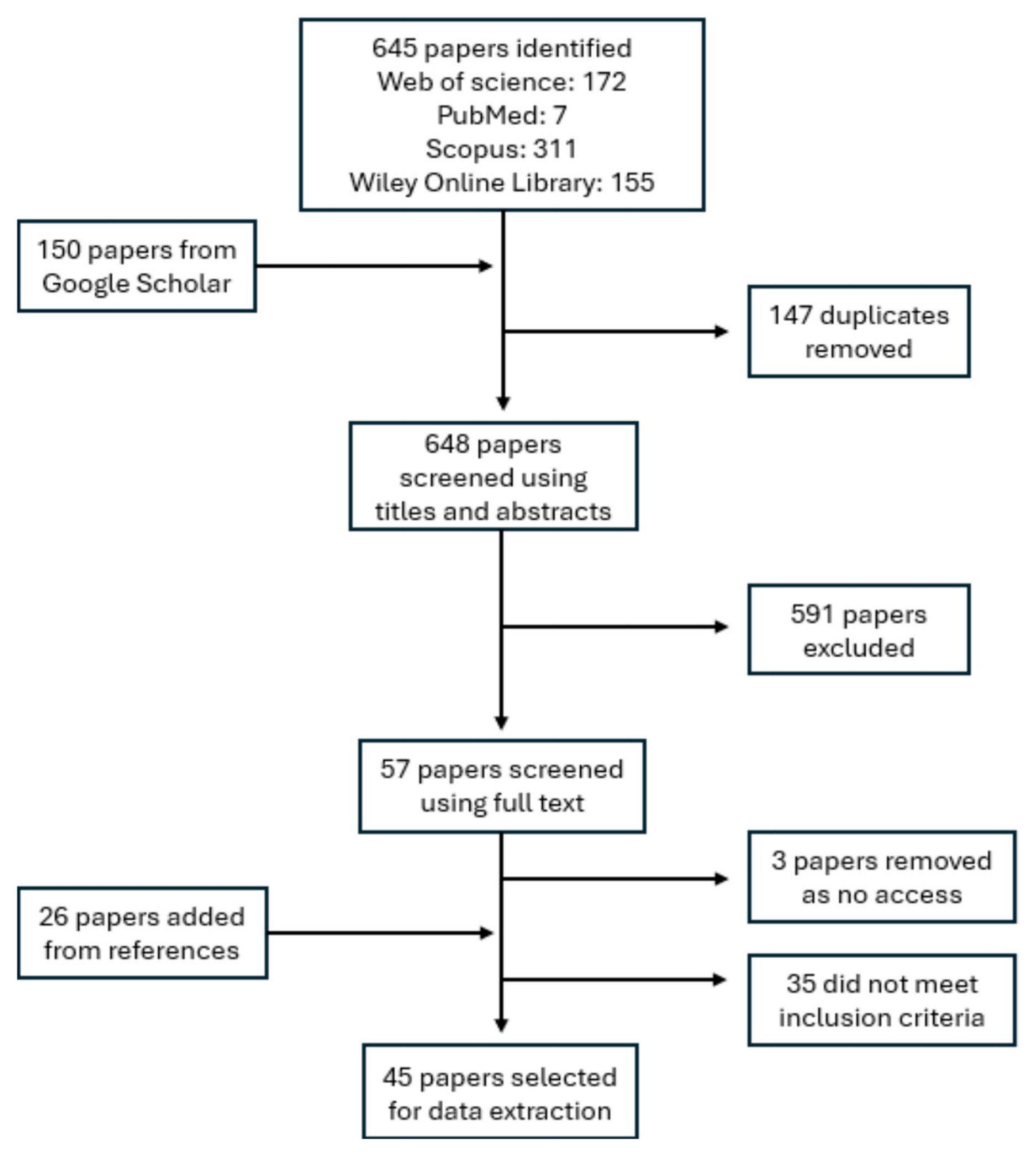
Flow diagram of the number of papers included and excluded from the literature search. 645 papers were identified from the four primary databases searched and 13 relevant papers were added from google scholar. After removing 147 duplicates, 648 papers remained for title and abstract screening. This resulted in 57 papers selected for full-text review. From the references of these papers, 26 further publications were added to the final section. Three papers were not accessible, and 35 documents did not meet the inclusion criteria after full text screening, resulting in 45 papers being used for data extraction

Figure 1 provides an overview of the search protocol and number of papers identified. An initial 645 papers were identified from the five databases searched. After removing duplicates, screening, excluding inaccessible papers (not available via institutional access, author contact, or the British Library), and incorporating relevant studies identified through reference lists, a total of 45 papers were included in the final selection.

### Study characteristics

**Fig. 2:**
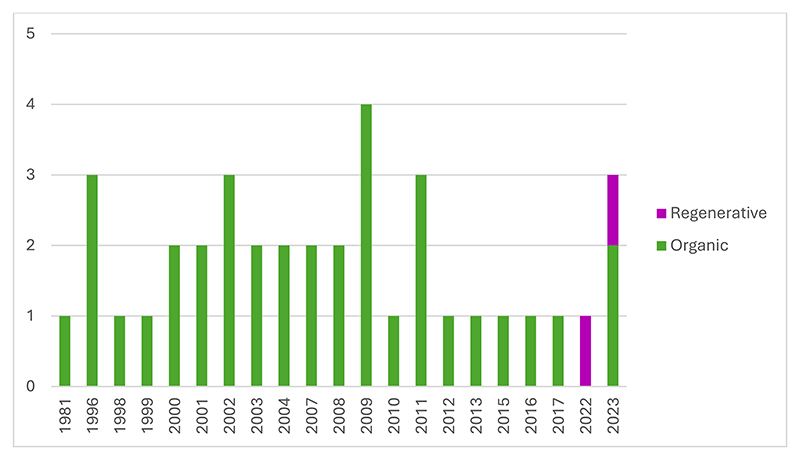
Bar chart displaying the number of documents published each year from 1981 to 2023, categorized by farming system: Organic (green) and regenerative (purple). Most documents relate to organic systems, with regenerative studies appearing from 2022

**Fig. 3:**
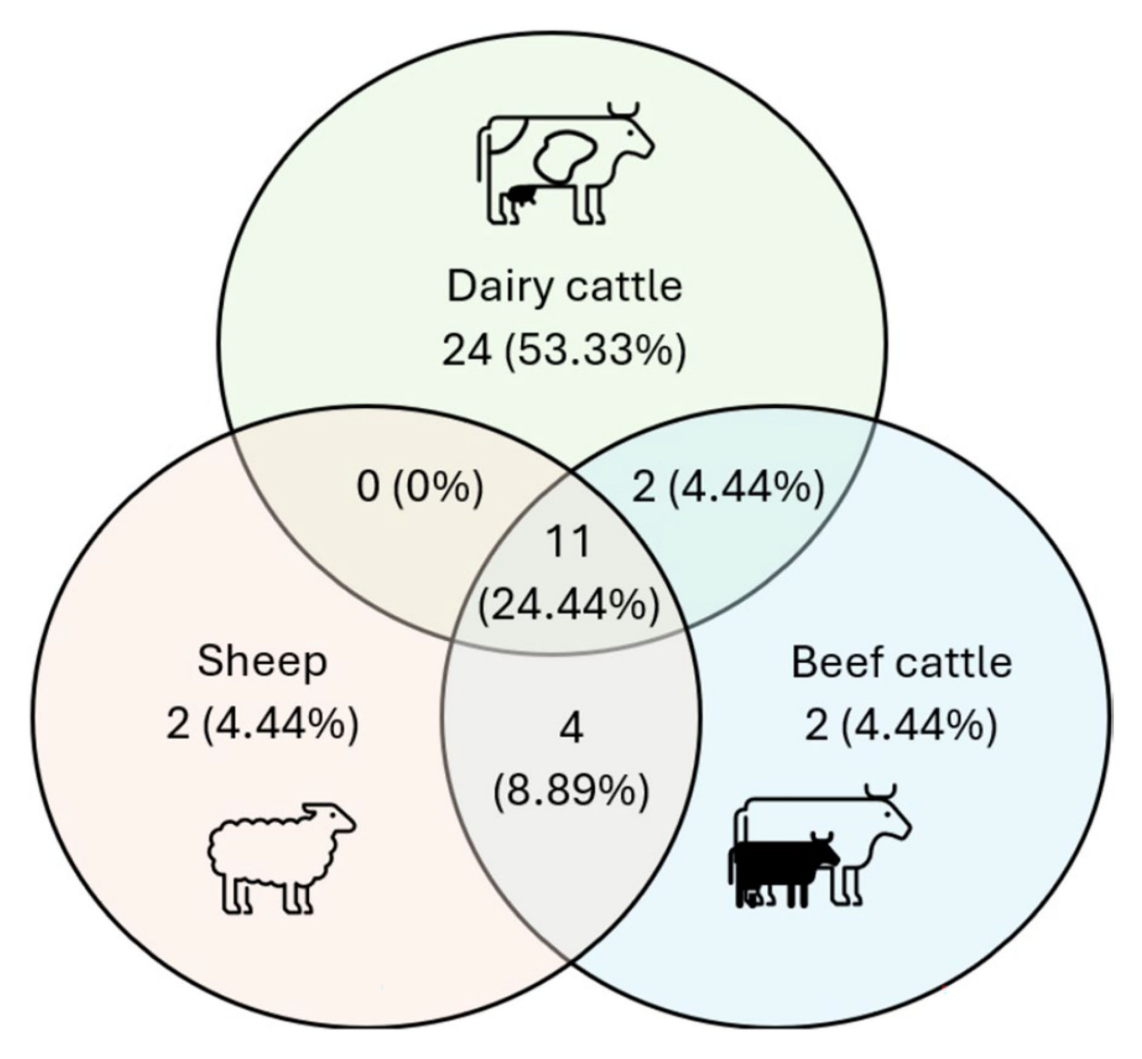
Venn diagram illustrating the focal species examined in regenerative and organic systems across the 45 documents included in this review. Over half of the studies focused on dairy cattle (53.33%), followed by studies examining all three species (24.44%). A smaller number of studies focused on sheep and beef (8.89%) and sheep (4.44%) or beef alone (4.44%). No studies looked at a combination of dairy cattle and sheep

Documents spanned the period 1981 to 2023 with publications on regenerative farming appearing post-2021 (Fig. 2). Out of the 45 papers, 43 (95.56%) studied organic and 2 (4.44%) regenerative farming. Over half of the livestock production systems studied were dairy cattle, followed by various combinations of beef, sheep and/or dairy, sheep only, and beef only (Fig. 3). Out of the 45 papers, 17 (37.78%) were conducted in the UK, 7 (15.56%) in England, 5 (11.11%) Scotland, 6 (13.33%) Wales, 6 (13.33%) England and Wales, and 4 (8.89%) Great Britain. Studies that took place in the UK or Great Britain either conducted across multiple countries studies or did not specify the location of the study.

Primary data were used in 36 papers (80.00%), secondary data in 2 papers (4.44%) (i.e., data collected for another purpose such as farm inspections) and the remaining 7 papers were reviews (15.56%). Of the 38 papers that used primary and secondary data, 34 (89.47%) reported evidence and 4 (10.53%) reported opinions on outcomes.

### Production management practices

More practices were identified in relation to organic farming (41 in total) than regenerative (9) (Table 2, Appendix 1). Four management practices were identified common to both farm systems: rotational grazing, grass-clover mixtures and home-grown or bought-in feed and bedding.

### Methods used in studies included

A total of 38 different methods were identified for collecting and analysing data on cattle and sheep farming to assess the outcomes of animal health, animal welfare, finances, productivity and biodiversity (Table 3). Of these methods, 13 (34.21%) were used to assess health, 7 (18.42%) finances, 5 (13.16%) welfare, 5 (13.16%) biodiversity, 2 (5.26%) productivity, and 6 (15.79%) could evaluate multiple outcomes. The most commonly used method for assessing health, as described by 8 papers, was milk sampling which was used to measure antibodies to helminth parasites, or evaluate somatic cell count or bactoscan count. Housing quality was the most commonly used method for assessing welfare, gross margin and net margin analysis for evaluating finances and the use of quadrats for assessing biodiversity. Fewer methods were identified evaluating productivity, although the most common metric was carcass conformation grading, which can also provide insights into health.

### Outcomes

All five outcomes were covered in documents on organic farms. Documents on regenerative farms were only identified assessing financial, biodiversity and productivity outcomes. Studies evaluating outcomes for all livestock species were found on organic farms, but no studies were found on regenerative dairy systems. All 7 review papers focused on organic farming: 4 addressed animal health and welfare, 2 reviewed biodiversity, and 1 covered farm finance.

Of the 38 studies that analysed primary and secondary data, 20 (52.63%) directly compared outcomes of organic and conventional farms and 1 study (2.63%) assessed farms before and after organic conversion. A further 6 studies (15.79%) undertook comparisons on a single farm type, such as assessing an outcome over time or the use of a particular practice, 5 papers (13.16%) described outcomes of one farm type without comparison and 1 paper (2.63%) undertook a combination of comparisons. The remaining 5 papers (13.16%) reported opinions on outcomes. None of the papers compared organic and regenerative farms to each other.

The outcomes of organic farms compared to conventional farms are shown in Table 4. Most of these studies found no difference in health and welfare outcomes when comparing organic and conventional farming. Among studies that reported effects, health outcomes were evenly split between positive and negative, while welfare outcomes leaned more towards positive effects with regards to cleanliness at housing and likeliness of compliance to welfare legislation. No studies found negative effects of organic farming on biodiversity, 1 paper found positive effects and 3 papers found no effect compared to conventional farming. The number of publications demonstrating increased financial performance of farming organically slightly outweighed the number finding decreased impacts, however in terms of productivity publications with worse performance (compared to conventional) dominated.

Studies undertaking comparisons on a single farm type are presented in Table 5. A range of comparisons were made including comparisons of management practices and outcomes at different life stages. Some conflicting results were found, for example the impact of cubicles on lameness and mastitis, and the impact of a self-sufficient or purchased feed system on productivity.

Studies reporting outcomes with no comparator (*n* = 5), such as benchmarking papers, predominantly reported on livestock health and welfare, and finances (Appendix Table 2). Only two of the studies with no comparator reported the same performance indicators, which were for milk yield. Opinion papers reported on a mix of perceived impacts of management practices, and perceived outcomes of particular farm systems. Perceptions of regenerative practices were all positive (Appendix Table 3). For organic farming, opinions of the impact of practices on welfare were predominantly positive; any management practices viewed to deteriorate welfare related to medication usage. Opinions on the health impacts of organic farming were varied; mixed grazing was perceived to benefit health yet diseases such as fly strike were still perceived to be a challenge.

## Discussion

This SLR aimed to identify the management practices used on regenerative and organic cattle and sheep farms in the UK and compare how practices and farming systems relate to outcomes of animal health and welfare, finances, productivity and biodiversity. A substantial evidence gap was identified in research assessing how regenerative livestock farming affects selected outcomes. The only paper identified assessed earthworm abundance as an indicator of soil health, highlighting a need for further research examining a broader range of outcomes. In contrast, a breadth of studies detailed the management practices used, and assessed the outcomes of organic farming, particularly in dairy cattle systems. The methods used to assess outcomes of organic agriculture offer valuable insights into study designs that could be used for research on regenerative systems. Additionally, the data collected in organic farming systems may serve as a useful comparator for future studies on alternative farming systems.

Although regenerative agriculture is a concept that has existed for more than four decades (Gabel 1979), it has only recently gained traction in the scientific literature resulting in a limited body of research in the UK. Despite this, the papers reviewed identified several management practices associated with regenerative livestock farms, including rotational grazing with herbal leys, integrated crop-livestock systems and cover crops, homegrown feed and bedding, no tillage, and methods to improve biodiversity, all of which align well with regenerative principles defined by farmer-led movements such as Groundswell (Ritz 2021). In contrast, in this SLR, there was a noticeable absence of studies reporting on medication usage, reproduction strategies or chemical inputs, although this may reflect study focus, rather than lack of use of a particular practice. Similar practices may be adopted to those undertaken on certified regenerative farms in Europe, such as a preference for homeopathy and limited use of antibiotics, which follow similar standards to organic but without rules on withdrawal periods that exceed legal guidelines (ROC 2023). This is likely to appeal to stakeholders who believe the restrictions on medication in organic systems challenge livestock welfare (Hovi and Kossaibati 2002), but may deter those concerned with antibiotic resistance, drug residues and maintenance of consumer trust.

The combination of these management practices, alongside no mention of supplementary feed, may be one explanation as to why no papers were found on regenerative dairy systems; it may be more challenging to implement regenerative management practices within dairy systems where highly productive animals with frequent milking requirements need more feed to produce large quantities of milk and maintain body condition. Despite these challenges, some farmers working for large dairy companies are piloting regenerative dairy production (Arla 2025; Yeo Valley 2025).

Management practices that may differentiate regenerative from organic farms, include the use of continuous grazing, mentioned in papers on organic farms but not regenerative, and the use of integrated crop-live-stock systems and no tillage, mentioned in papers on regenerative farms but not organic. Additionally, management practices relating to actively encouraging biodiversity, such as planting trees, were only mentioned in papers on regenerative agriculture, contrasting with organic where the focus is to prohibit practices which cause further decline. Furthermore, while not identified within this review, reduced, rather than strict exclusion of synthetic herbicides and pesticides may also be a key differentiating factor, although opinions appear to be divided on whether the use of synthetic inputs is compatible with regenerative principles (Markowicz 2024; Newton et al. 2020). Despite these differences, the evidence gathered within this review suggests that it may be possible for an organically certified farm to also be considered regenerative; certifications offered by the Regenerative Organic Alliance demonstrate this (ROC 2023). The lack of studies directly comparing regenerative and organic farms may point to the fact that the differences between these two approaches are not yet clearly defined. Differences relating to biodiversity encouragement may diminish over the coming years as subsidy schemes and net-zero commitments align with proactive measures to increase biodiversity, such as increasing tree cover, hence it is likely that most farming systems will adopt these practices going forward.

The effect of farming system on outcomes of animal health and welfare, biodiversity, finances and productivity could only be evaluated on organic farms as no studies compared regenerative farms to other farming systems and only against conventional systems. Results from studies on organic farms indicate slightly better financial performance than on conventional farms, but a decline in productivity, predominantly due to reductions in concentrate feed and restrictions on medication use. Findings demonstrate that financial improvement is likely due to a reduction in inputs such as fertilisers and herbicides and higher sales prices for organic meat although this review has demonstrated that concentrate feed and other inputs are still commonplace on organic farms. Despite identifying a positive perception of the impact of organic farming on animal health and welfare, most papers evaluating these outcomes found no difference between organic and conventional farms. However, the heterogeneity of evidence gathered within this review means that it is challenging to draw conclusions about health and welfare outcomes as a large variety of management practices and outcomes were measured. Findings of this review suggest that improved animal health and welfare may relate to improvements in housing, space and feed, but that stricter medication rules and reduced feed availability at similar stocking rates to conventional may hinder health and welfare. Similarly, a key goal of organic agriculture is to improve biodiversity, yet this was not demonstrated within this review unlike in prior research which encompass a broader range of farming systems, including arable systems (Hole et al. 2005; Stein-Bachinger et al. 2021). It is also possible that the differences between organic and conventional systems are more prominent, and more commonly researched, on arable farms where significant differences in management practices are observed, such as the lack of synthetic chemicals.

Interviews undertaken by Jordon et al. (2022) suggest a belief among farmers and industry representatives that regenerative practices positively affect outcomes of animal health, farm business, productivity and biodiversity. However, this review only identified evidence of improved earthworm abundance, as reported by Trickett and Warner (2022) as an indicator of soil health. This may be due to management practices only being deemed as regenerative now, and not linked to the term in the past, selective reporting of management practices within studies and the topic having not yet attracted substantial research activity in UK cattle and sheep system. Although regenerative agriculture is grounded in principles that focus on soil health (Ritz 2021), research should measure outcomes relating to broader goals of both arable and livestock farming. Further research into the outcomes of regenerative agriculture would be valuable to a range of stakeholders, including customers who may pay for regenerative products based on assumed benefits, and for farmers who may take risks to transition to regenerative agriculture without scientific evidence of the outcomes.

This review demonstrates that the breadth of study designs and methods used to assess outcomes of livestock farms can make it difficult to draw definitive conclusions about how farming systems perform and compare. Due to this variety, a structured quality assessment was deemed too restrictive for the purpose of this review and therefore was not undertaken. Additionally for this reason, a formal assessment of risk of bias due to missing results was not undertaken. Risk of bias was minimised by including grey literature, and both statistically significant and non-significant results. The number of papers on organic farming, along with the research standard of comparing organic to conventional, reflects its position as the dominant ‘alternative’ to conventional agriculture, despite only 3% of cattle and 2.2% of sheep being raised organically in the UK (Defra Organic Farming Statistics 2024). Unlike regenerative systems, organic farming is clearly defined and certified, making it more straightforward for researchers to study. Furthermore, the association of organic food with natural processes, animal welfare and care for the environment (Shafie & Rennie 2012) has fueled research interest in empirically determining whether this is the case. To understand how farms compare, and the trade-offs within each system, it may be beneficial to design future studies that allow comparisons to be made with existing findings of studies on organic and conventional farms, either applying or not applying regenerative principles.

## Conclusion

This SLR illustrated a significant gap in research on regenerative systems in UK cattle and sheep farming systems, contrasting with the relatively well-documented management practices and outcomes on organic farming. Overall, this review provides a useful benchmark for transitions to more sustainable farming systems in coming years. Research on organic farming demonstrated that, compared to conventional farming, improved financial performance is traded off for productivity, and the impact on animal health, animal welfare and biodiversity is variable. Furthermore, this research demonstrated that assessing and comparing the outcomes of different farming systems in the UK can be a challenge, due to the diversity of methods used, variety of outcomes measured and a general lack of standardisation in onfarm research. Further research, utilising commonly used study designs and methods identified within this review may offer a useful starting point for creating comparable evidence of outcomes across different livestock farms.

## Supplementary Material

**Supplementary Information** The online version contains supplementary material available at https://doi.org/10.1007/s13165-026-00559-3.

Appendix 1

Appendix 2

Appendix 3

## Figures and Tables

**Table 1:** Phrases and words used in final search term

Farming System	Location	Population	Outcome	Exclude
organic OR "regenerative agriculture" OR "regenerative farm*"	"United Kingdom" OR England OR Wales OR Scotland OR "Northern Ireland" OR British OR Britain OR "Great Britain"	sheep OR ewe* OR ovine OR ram* OR lamb* OR cow* OR cattle OR livestock OR calf OR calves OR bull* OR heifer* OR bovine* OR dairy OR beef OR suckler	health OR disease OR infection OR virus OR welfare OR biodivers* OR wildlife OR econom* OR financ* OR cost OR productiv* OR performance	"New South Wales" OR "British Columbia"

**Table 2:** Management practices used on regenerative and organic beef (B), sheep (S) and dairy (D) farms in the UK and counts of the number of papers that mentioned each practice (shown as numbers after the letters). Colours indicate the percentage of papers within each category mentioning each practice (shown as numbers after the letters). Colours indicate the percentage of papers within each category mentioning each practice

Percentage	Colour
0	
1-25	
26-50	
51-75	
76-99	
100	

Category	Practice	Regenerative	Organic
Grazing management	Rotational grazing	B2, D0, S1	B3, D4, S3
	Continuous grazing		B0, D1, S0
	Mixed grazing		B4, D1, S6
	Integrated crop-livestock	B2, D0, S1	
Stocking density	Low		B5, D4, S3
Pasture composition	Grass – clover mixtures	B1, D0, S0	B2, D5, S2
	Herbal leys	B1, D0, S1	
Feed	Concentrates		B7, D8, S3
	Fodder		B1, D1, S0
	Conserved forage (hay and silage)		B5, D6, S5
	Forage and roughage		B1, D3, S1
	Straw		B1, D1, S1
	Organic feed		B0, D1, S0
Minerals and supplements	Seaweed meal		B1, D2, S0
	Mineral supplements		B2, D2, S0
	Protein supplements		B0, D0, S1
Housing	Winter housing		B5, D12, S0
	Bedding in indoor housing		B1, D1, S1
	Outwintered		B2, D0, S2
Health management	Preventative health strategies not routine drugs		B0, D0, S1
	Alternative remedies (e.g. herbal treatments, homeopathy)		B0, D10, S2
	Foot baths		B1, D2, S2
	Foot trimming		B0, D2, S0
	Pharmaceutical Medication (e.g. fenbendazole, antibiotics)		B2, D10, S3
	Vaccinations		B1, D4, S3
Reproduction	All year-round calving/lambing		B0, D3, S0
	Summer calving/lambing		B1, D1, S0
	Spring calving/lambing		B4, D1, S3
	Autumn calving/lambing		B0, D1, S0
	Winter weaning		B2, D0, S0
	Summer weaning		B0, D0, S1
	Early weaning <6 weeks		B0, D1, S0
	Late weaning >10 weeks		B1, D1, S0
	Natural milk for young		B1, D1, S0
	Artificial insemination		B2, D3, S0
	Natural service		B2, D2, S0
	Birthing indoors		B0, D0, S3
	Birthing outdoors		B1, D0, S2
	Single and twin rearing livestock graze separately		B0, D0, S2
Inputs – feed and bedding	Brought in	B1, D1, S0	B0, D1, S2
	Produced on farm	B1, D0, S1	B1, D1, S1
Tillage	No tillage	B1, D0, S0	
Fertilisers	Organic fertilisers		B1, D1, S1
Milking	Standard milking frequency – twice per day		D1
Biodiversity	Integrate trees on farm	B1, D0, S1	
	Maintain and plant hedgerows	B1, D0, S1	

**Table 3:** Methods used to research the outcomes of animal health and welfare, productivity and finances, and biodiversity on organic and regenerative cattle and sheep farms. Methods are grouped by their primary outcome focus, although many can be applied across multiple outcomes

Species applicable	Data collection method	Purpose	References
**Health/physical wellbeing**			
Cattle and sheep	Faecal sampling	Identify helminth eggs, lung worm and liver fluke	(Ellis et al. 2011; Jackson et al. 2017; Maggs et al. 2008; Mitchell et al. 2010; Weller and Cooper 1996a)
	Blood sampling	Assess plasma proteins, albumin, globulin and serum pepsinogen concentrations to monitor gastrointestinal parasite infections	(Ellis et al. 2011; Jackson et al. 2017)
	Pasture sampling and larval counts	Establish presence and load of parasites on pasture	(Ellis etal. 2011)
	Body condition scoring	Assess an animal’s fat and muscle reserves	(Adamson 2002; Huxley et al. 2003, 2004; Keatinge 2001; Langford et al. 2011; Rutherford et al. 2016)
	Coat/fleece condition	Evaluate overall health, nutrition and grooming	(Huxley et al. 2003, 2004)
	Signs of injury or trauma	Monitor physical harm or stress	(Huxley et al. 2003, 2004)
	Data taken from animal health inspections	Inform on health history	(KilBride et al. 2012)
	Cleanliness	Scores given to determine the cleanliness of various body regions e.g. flanks, hind legs, tail and udder. Also an indicator of environmental conditions. animal comfort, health and hygiene	(Ellis et al. 2007; Huxley et al. 2003, 2004; Rutherford et al. 2008)
Cattle	Individual and herd milk sampling	Establish exposure to parasites, evaluate somatic cell count or bactoscan count	(Ellis et al. 2007. 2011; Haskell et al. 2009; Hovi and Roderick 2000; Weller and Bowling 2000; Weller and Cooper 1996a, 1996b; Weller and Davies 1998)
	Claw condition	Identify issues relating to poor housing or disease	(Huxley et al. 2003, 2004)
	Rumen fill	Assess recent feed intake and digestive status	(Huxley et al. 2003, 2004)
	Hock condition scoring	Monitor problems such as lameness, joint infections. or poor housing conditions	(Rutherford et al. 2008)
	Assessments of rising restrictions	Evaluate ease of standing	(Huxley et al. 2003, 2004)
	Milk blood β-hydoxybutyrate (BHBA) measurements using dipstick keto-test	Inform on animal health and welfare as BHBA increases when animals are not fed enough to meet energy requirements	(Rutherford et al. 2016)
	Weight	Evaluate daily weight gain for assessing health and productivity	(Adamson 2002; Frost et al. 2009; Jackson et al. 2017; Keatinge 2001)
**Welfare**			
Cattle and sheep	Flight distance	Determine calmness and human bond	(Huxley et al. 2003, 2004)
	Observations of aggressive interactions	Identify signs of stress	(Langford et al. 2011)
	Observations of the number of animals grooming	Identify positive welfare (e.g. affiliative bonds. comfort) or negative welfare (e.g. stress)	(Langford et al. 2011)
	Quality of winter housing (measurements, cleanliness. flooring etc.)	Assess space, comfort and cleanliness	(Langford et al. 2009; Weller and Cooper 1996a)
Cattle	Observations of the number of cows idling	Identify potential discomfort, illness, or poor environmental conditions	(Huxley et al. 2003, 2004)
	Observations of the number of cows ruminating	Assess gut functionality or identify positive welfare (usually performed by healthy, relaxed, unstressed cattle)	(Langford et al. 2011)
**Financial**			
Cattle and sheep	Gross margin analysis	Measure the revenue retained after accounting for the cost of producing the goods and services sold	(Fowler et al. 2001; Frost et al. 2009; Keatinge 2001; Shell and Younie 2001)
	Family farm income	Assess the income from the farmer and spouse, and investors in the business	(Tranter et al. 2007; Vine and Bateman 1981)
	Net margin/net profit/net farm income	Calculate profits by accounting for all business expenses during a given period. This includes the cost of goods sold and operating/variable costs	(Fowler et al. 2001; Frost et al. 2009; Vine and Bateman 1981; Wilson 2011)
	TIPI-CAL simulation model	Model-based assessment to stimulate the effect of policy and technological changes on farm profitability	(Häring 2003)
	Management and investment income	Measure total farm enterprise outputs less total inputs (including the value of the labour input of the farmer and spouse)	(Fowler et al. 2001)
	Cash income	Calculate the difference between receipts and expenditure on current account, before depreciation charges and investment spending	(Fowler et al. 2001)
	Cost of production data	Calculate the cost of producing 1 kg of beef/lamb. Includes variable, forage and fixed costs, as well as imputed costs of production including unpaid labour (farmer/spouse/other), imputed rent (rental equivalent) and interest on tenant’s capital (the interest on capital items such as livestock, machinery and buildings	(Frost et al. 2009)
**Productivity**			
Cattle and sheep	Carcass weight	Measure meat production per animal. Can be combined with age of slaughter as a measure of daily weight gain	(Shell and Younie 2001)
	Carcass conformation grading	Assess visually the overall weight, shape and flesh coverage of the carcase	(Adamson 2002; Keatinge 2001)
**Biodiversity**			
N/A	Quadrats/grids	Used to record flora or fauna within a defined area, or to identify the distribution and condition of land habitat types	(Adamson 2002; Fraser et al. 2013; Keatinge 2001)
	Transects	Sample presence, abundance or distribution of specific vegetation types along a line across a habitat	(Adamson 2002)
	Earthworm counts	Inform on soil health and earthworm abundance	(Trickett and Warner 2022)
	Dung beetle presence and abundance	Used as an indicator species to inform on biodiversity and ecosystem health	(Beynon et al. 2015)
	Arial photography and GIS	Assess vegetation cover	(Keatinge 2001)
**Social science and farm records**			
Cattle and sheep	Questionnaire or survey of farmers and advisers	Collect information on opinions, management practices and pre-measured outcomes	(Borelli et al. 2023; Chylinski et al. 2023; Hovi and Kossaibati 2002; Langford et al. 2009; Roderick et al. 1996, 1999)
	Interviews of farmers and industry representatives	Gain insights into farm management, pre-recorded outcomes, financials, management practices and perceived benefits and drawbacks of farming practices. To rate perceived disease impacts	(Chylinski et al. 2023; Ellis et al. 2007; Hovi and Roderick 2000; Jordon et al. 2022; Langford et al. 2009, 2009; Roderick et al. 1999; Rutherford et al. 2008; Vine and Bateman 1981)
	Analysis of farm records	Gain information on disease events, percentage of animals affected, culling rates, financial loss from disease	(Adamson 2002; Hovi and Roderick 2000; Weller and Bowling 2000)

**Table 4:** The outcomes of organic farming compared to conventional farming (positive, same, negative) on beef, dairy and sheep farms including types of effects assessed and statistical significance

Impact of outcome on organic compared to conventional	Positive impact	No impact	Negative impact
Species	Health		
Dairy	- Decrease in hock lesions and swellings*** - Decrease in lameness prevalence*, *n* = 80 (Rutherford et al. 2008)	Overall health and fertility, n = 11 (Weller and Cooper 1996a)	
	
		Overall health (disease incidence, culling rates)^M^,*n* = 80 (Langford et al. 2009) Somatic cell counts, *n =* 80 (Haskell et al. 2009) Overall health, *n* = 11 (Weller & Cooper 1996b) Prevalence of thin cows^NS^, *n* = 43 (Rutherford et al. 2016) Faecal egg counts^NS^, *n* = 11 (Maggs et al. 2008)	
Beef, dairy		Mastitis risk^M^, *n* = 23 (Hovi & Roderick 2000)	
Sheep	Decreased anthelmintic resistance, *n* = 122 (Mitchell et al. 2010)	Body condition scores, *n = 4* (Adamson 2002)	Decrease in body condition scores since organic conversion, *n =* 1 (Frost et al. 2009) Body condition score^M^, *n =* 3 (Keatinge 2001)
	**Welfare**		
Dairy	Improved cleanliness at housing***. *n =* 14 (Ellis et al. 2007)	Behaviour (interactions, aggression, feeding motivation)^NS^, *n* = 40 (Langford et al. 2011) Cleanliness^NS^, *n* = 80 (Langford et al. 2009) Cleanliness at pasture^NS^, *n* = 14 (Ellis et al. 2007)	
Beef, dairy, sheep	Reduced risk of non-compliance to welfare legislation*, *n* = 38.651 (KilBride et al. 2012)		
	**Finances**		
Sheep			Decreased gross margin, *n* = 4 (Adamson 2002)
Dairy		Financial performance ^NS^, *n* = 228 (Wilson 2011)	
Beef. sheep	Increased financial performance (net farm income, whole farm income and gross margin)^NS^, n= 100 (Frost et al. 2009) Increased financial performance (Price per animal, gross margin)^NS^, *n* = 3 (Keatinge 2001)		
Beef. Dairy. Sheep	Increase in family farm income (Tranter et al. 2007), *n* = 27		Decreased financial performance (net farm income and gross margin), *n* = 30 (Vine and Bateman 1981)
	**Productivity**		
Dairy		Growth curves and daily live weight gain, *n* = 3 (Jackson et al. 2017)	Decreased milk yield, *n* = 80 (Haskell et al. 2009) Decreased milk yield***, *n* = 80 (Langford et al. 2009) Decreased milk yield, *n* = 80 (Rutherford et al. 2009)
Sheep			- Decrease in reproductive performance (number of lambs per ewe, number of barren lambs) - Decrease in ewe liveweight* - Decreased growth rate in twin lambs*, *n* = 4 (Adamson 2002) Decrease in ewe liveweight since organic conversion ^NS^, *n* = 1 (Frost et al. 2009) - Decreased ewe and lamb live-weight*** - Ewes carried fewer twins**, *n* = 3 (Keatinge 2001)
	**Biodiversity**		
Beef, dairy	Improved dung beetle diversity, *n* = 18 (Beynon et al. 2015)		
Beef, dairy, sheep		Habitat diversity, grassland and plant species diversity and richness^M^, *n* = 45 (Fraser et al. 2013)	
Sheep		Botanical composition^NS^, *n* = 3 (Keatinge 2001) Vegetation dynamics, *n* = 26 (Adamson 2002)	

^M^(A mix of significant and non-significant results, and overall conclusion as stated by author reported), ^NS^*p* > 0.05, *p < 0.05, **p < 0.01, ***p < 0.001. No superscript indicates no statistical test was reported in the study. *N* = the experimental unit

**Table 5:** Results of comparisons of management practices undertaken on beef (B), dairy (D) and sheep (S) organic and regenerative farms

Farming system	Comparator 1	Comparator 2	Results	Reference
Organic	**Health and welfare**			
	First season grazing animals	Second season grazing animals	First season grazing animals experienced significant parasite burdens, which rose during the second half of the grazing season**, *n* = 24	(Ellis et al. 2011)^D^
	Housed in yards	Housed in cubicles	Lower lameness incidence in yards, *n* = 11	(Weller and Cooper 1996b)^D^
	Housed in cubicles in sheds	Housed in straw yards	Lower mastitis incidence in cubicle sheds, *n* = 10	(Weller and Bowling 2000)^D^
	Lactations 1 to 4	Lactations 5–9 +	Somatic cell counts increased sharply after lactation 4, *n* = 183	(Weller and Davies 1998)^D^
	High quality records in herd health plan	Low quality records in herd health plan	No link between the herd health plan and quality of records, and animal health and welfare, *n* = 15	(Huxley et al. 2003)^D^
	**Productivity**			
	Self-sufficient system: A cropping strategy to provide forage and cereal concentrates	Purchased feed system: forage produced on farm and purchased concentrate feed (barley, wheat, field beans, soybeans, stocking density of 1.6 cows/ha.)	- The self-sufficient system had improved milk production from forage - Peak yields were similar in the self-sufficient and purchased-feed system but high yield did not persist in the self-sufficient system - Pregnancy rate was lower in the self-sufficient system (81.7 vs 89.8%) - Total milk yield was higher in the purchased-feed system than the SS system *n* = 2	(Weller and Bowling 2004)^D^
Regenerative	**Biodiversity**			
	Zero tillage, and permanent grassland	Zero tillage + mob grazing	Zero tillage combined with mob grazing increased earthworm abundance and epigeic species*, *n* = 3	(Trickett and Warner 2022)^B^

^NS^*p* > 0.05, *p < 0.05, **p < 0.01, ***p < 0.001. No superscript indicates no statistical test was undertaken. *N* = the experimental unit

## Data Availability

No datasets were generated or analysed during the current study.

## References

[R1] Accredited Official Statistics Summary (2025). GOV.UK.

[R2] Adamson (2002). Defra Final project report: Organic beef and sheep production in the uplands.

[R3] Arla (2025). Regenerative Dairy Farming How dairy farms can contribute to the fight against climate change and biodiversity loss.

[R4] Beynon S, Wainwright W, Christie M (2015). The application of an ecosystem services framework to estimate the economic value of dung beetles to the UK cattle industry. Ecol Entomol.

[R5] Borelli E, Ellis K, Pamphilis N, Tomlinson M, Hotchkiss E (2023). Factors influencing Scottish dairy farmers’ anti-microbial usage, knowledge and attitude towards anti-microbial resistance. Prev Vet Med.

[R6] (2024). Chapter 9: Intermediate consumption.

[R7] Chylinski C, Athanasiadou S, Thueer S, Grovermann C, Moakes S, Hoste H, Petkevicius S, Verwer C, Verkaik J, Werne S (2023). Reducing anthelmintic inputs in organic farming: are small ruminant farmers integrating alternative strategies to control gastrointestinal nematodes?. Vet Parasitol.

[R8] Department for Environment, Food & Rural Affairs (2020). The path to sustainable farming: An agricultural transition plan 2021 to 2024.

[R9] Department for Environment, Food and Rural Affairs (2024). Organic farming statistics 2024: United Kingdom.

[R10] Ellis KA, Innocent G, Mihm M, Cripps P, McLean WG, Howard CV, Grove White D (2007). Dairy cow cleanliness and milk quality on organic and conventional farms in the UK. J Dairy Res.

[R11] Ellis KA, Jackson A, Bexiga R, Matthews J, McGoldrick J, Gilleard J, Forbes AB (2011). Use of diagnostic markers to monitor fasciolosis and gastrointestinal nematodes on an organic dairy farm. Vet Rec.

[R12] (2024). Farming Evidence Pack: A high-level overview of the UK agricultural industry.

[R13] (2025). Farming evidence—Key statistics (accessible version).

[R14] Fowler S, Wynne-Jones I, Lampkin N (2001). Organic farm incomes in England and Wales 1998/99.

[R15] (2023). Framework for Regenerative Organic Certified.

[R16] Fraser M, Vale J, Firbank L (2013). Effect on habitat diversity of organic conversion within the less favored areas of England and Wales. Agroecol Sustain Food Syst.

[R17] Frost D, Morgan M, Moakes S (2009). A farmer’s guide to organic upland beef and sheep production (Organic farming technical guide).

[R18] Gabel M (1979). Ho-Ping: Food for Everyone.

[R19] Government food strategy (2022). GOV.UK.

[R20] Haddaway NR, Collins AM, Coughlin D, Kirk S (2015). The role of Google Scholar in evidence reviews and its applicability to grey literature searching. PLoS One.

[R21] Häring AM (2003). Organic dairy farms in the EU: production systems, economics and future development. Livest Prod Sci.

[R22] Haskell M, Langford F, Jack M, Sherwood L, Lawrence A, Rutherford K (2009). The effect of organic status and management practices on somatic cell counts on UK dairy farms. J Dairy Sci.

[R23] Hole DG, Perkins AJ, Wilson JD, Alexander IH, Grice PV, Evans AD (2005). Does organic farming benefit biodiversity?. Biol Conserv.

[R24] Hovi M, Kossaibati M (2002). The impact of organic standards on cattle health and welfare—a survey of veterinarians, advisors and inspectors. Cattle Pract.

[R25] Hovi M, Roderick S (2000). Mastitis and mastitis control strategies in organic milk. Cattle Pract.

[R26] Hughes KM, Kaiser MJ, Jennings S, McConnaughey RA, Pitcher R, Hilborn R, Amoroso RO, Collie J, Hiddink JG, Parma AM, Rijnsdorp A (2014). Investigating the effects of mobile bottom fishing on benthic biota: a systematic review protocol. Environ Evid.

[R27] Huxley J, Burke J, Roderick S, Main DCJ, Whay HR (2004). Animal welfare assessment benchmarking as a tool for health and welfare planning in organic dairy herds. Vet Rec.

[R28] Huxley J, Burke J, Roderick S, Main D, Whay H (2003). Herd health and welfare benchmarking on organic dairy farms in South-West England. Cattle Pract.

[R29] Jackson A, Ellis KA, McGoldrick J, Jonsson NN, Stear MJ, Forbes AB (2017). Targeted anthelmintic treatment of parasitic gastroenteritis in first grazing season dairy calves using daily live weight gain as an indicator. Vet Parasitol.

[R30] Jordon MW, Willis KJ, Bürkner P-C, Petrokofsky G (2022). Rotational grazing and multispecies herbal leys increase productivity in temperate pastoral systems – a meta-analysis. Agric Ecosyst Environ.

[R31] Keatinge R (2001). Organic beef and sheep production (OF0147).

[R32] KilBride AL, Mason SA, Honeyman PC, Pritchard DG, Hepple S, Green LE (2012). Associations between membership of farm assurance and organic certification schemes and compliance with animal welfare legislation. Vet Rec.

[R33] Langford FM, Rutherford KM, Jack MC, Sherwood L, Lawrence AB, Haskell MJ (2009). A comparison of management practices, farmer-perceived disease incidence and winter housing on organic and non-organic dairy farms in the UK. J Dairy Res.

[R34] Langford FM, Rutherford K, Sherwood L, Jack M, Lawrence A, Haskell M (2011). Behavior of cows during and after peak feeding time on organic and conventional dairy farms in the United Kingdom. J Dairy Sci.

[R35] (2025). Livestock populations in the United Kingdom at 1 June 2025.

[R36] Maggs L, Athanasiadou S, Sherwood L, Haskell M (2008). Levels of parasitism on organic and non-organic dairy farms in Scotland. Vet Rec.

[R37] Markowicz M (2024). Regenerative farming practices and their impact on the soil health.

[R38] McCain Foods (2024). McCain’s regenerative agriculture framework.

[R39] Microsoft Corporation (2025). Microsoft Excel (Version 2412) [Computer software].

[R40] Mitchell ESE, Hunt KR, Wood R, McLean B (2010). Anthelmintic resistance on sheep farms in Wales. Vet Rec.

[R41] (2024). Nestlé Agriculture Framework.

[R42] Newton P, Civita N, Frankel-Goldwater L, Bartel K, Johns C (2020). What is regenerative agriculture? A review of scholar and practitioner definitions based on processes and outcomes. Front Sustain Food Syst.

[R43] Ouzzani M, Hammady H, Fedorowicz Z, Elmagarmid A (2016). Rayyan—A web and mobile app for systematic reviews. Systematic Reviews.

[R44] Page MJ, McKenzie JE, Bossuyt PM, Boutron I, Hoffmann TC, Mulrow CD, Shamseer L, Tetzlaff JM, Akl EA, Brennan SE, Chou R (2021). The PRISMA 2020 statement: An updated guideline for reporting systematic reviews. PLOS Medicine.

[R45] Reed J, Deakin L, Sunderland T (2015). What are ‘integrated landscape approaches’ and how effectively have they been implemented in the tropics: a systematic map protocol. Environ Evid.

[R46] (2026). Regenerative Farmers of UK Map.

[R47] Regenerative Grazing (2025). Yeo Valley regenerative farming.

[R48] Ritz K (2021). Principles of regenerative agriculture.

[R49] Roderick S, Hovi M, Short N (1999). Animal health and welfare issues on organic livestock farms in the UK: results of a producer survey. BSAP Occas Publ.

[R50] Roderick S, Shart N, Hovi M (1996). Animal health and welfare research priorities. Organic Livestock Production.

[R51] Roe D, Fancourt M, Sandbrook C, Sibanda M, Giuliani A, Gordon-Maclean A (2014). Which components or attributes of biodiversity influence which dimensions of poverty?. Environ Evid.

[R52] Rutherford KMD, Langford FM, Jack MC, Sherwood AB, Lawrence AB, Haskell MJ (2016). Organic dairy cow management and indicators of energy balance. Vet Rec.

[R53] Rutherford Langford F, Jack M, Sherwood L, Lawrence A, Haskell M (2008). Hock injury prevalence and associated risk factors on organic and nonorganic dairy farms in the United Kingdom. J Dairy Sci.

[R54] Rutherford LF, Jack M, Sherwood L, Lawrence A, Haskell M (2009). Lameness prevalence and risk factors in organic and non-organic dairy herds in the United Kingdom. Vet J.

[R55] (2026). SFI26: Details, definitions and what to expect – The farming blog.

[R56] Shafie FA, Rennie D (2012). Consumer perceptions towards organic food. Procedia - Soc Behav Sci.

[R57] Shell D, Younie D, Wilkinson J, Younie D (2001). Organic livestock farming.

[R58] Soil Association (2024). Organic standards for Great Britain.

[R59] Stein-Bachinger K, Gottwald F, Haub A, Schmidt E (2021). To what extent does organic farming promote species richness and abundance in temperate climates? A review. Org Agric.

[R60] Tittonell P, El Mujtar V, Felix G, Kebede Y, Laborda L, Luján Soto R, de Vente J (2022). Regenerative agriculture—agroecology without politics?. Front Sustain Food Syst.

[R61] Tranter R, Holt G, Grey P (2007). Budgetary implications of, and motives for, converting to organic farming: case study farm business evidence from Great Britain. Biol Agric Hortic.

[R62] Trickett T, Warner D (2022). Earthworm abundance increased by mob-grazing zero-tilled arable land in South-East England. Earth.

[R63] Uberoi E (2023). English farm statistics: Challenges, farm types and regions.

[R64] Unilever (2020). Unilever climate & nature fund.

[R65] United Nations (2023). Sustainable development goals: 17 goals to transform our world.

[R66] Vine A, Bateman D (1981). Organic farming systems in England and Wales: Practice, performance and implications.

[R67] Weller RF, Bowling PJ (2000). Health status of dairy herds in organic farming. Vet Rec.

[R68] Weller RF, Bowling PJ (2004). The performance and nutrient use efficiency of two contrasting systems of organic milk production. Biological Agriculture.

[R69] Weller RF, Cooper A (1996a). Conversion to organic milk production.

[R70] Weller RF, Cooper A (1996b). Health status of dairy herds converting from conventional to organic dairy farming. Veterinary Record.

[R71] Weller RF, Davies DW (1998). Somatic cell counts and incidence of clinical mastitis in organic milk production. Vet Rec.

[R72] Wilson P (2011). Decomposing variation in dairy profitability: the impact of output, inputs, prices, labour and management. J Agric Sci.

