# Supplementary material for: Outcomes of management practices on regenerative and organic cattle and sheep farms in the UK—a systematic literature review
Source: Org Agric. Author manuscript; Available in PMC 2026 Aug 15. (PMC7619368; doi:10.1007/s13165-026-00559-3)
Supplement: Supplementary file 1 [file EMS216180-supplement-Appendix_1.docx]

Appendix 2. Results of reports on organic farms that did not undertake comparisons. B = Beef, D = Dairy, S = Sheep.

| Outcome | Aspect of livestock farming assessed | Outcome | Reference |
| --- | --- | --- | --- |
| Health and welfare | Livestock in herd/flock with anthelmintic resistance (%) | S = 24 | (Chylinski et al., 2023) |
|  | Livestock thin (%) | D = 2.8 | (Huxley et al., 2004) |
|  | Livestock fat (%) | D = 7.1 | (Huxley et al., 2004) |
|  | Livestock with bloated rumen (%) | D = 35.7 | (Huxley et al., 2004) |
|  | Livestock with hollow rumen (%) | D = 0 | (Huxley et al., 2004) |
|  | Livestock lame (%) | D = 24.2 | (Huxley et al., 2004) |
|  | Livestock with claw overgrowth (%) | D = 30.8 | (Huxley et al., 2004) |
|  | Livestock with claw conformation (%) | D = 0 | (Huxley et al., 2004) |
|  | Livestock with dirty hindlimbs (%) | D = 100 | (Huxley et al., 2004) |
|  | Livestock with dirty udders (%) | D = 40 | (Huxley et al., 2004) |
|  | Livestock with dirty flanks (%) | D = 33.3 | (Huxley et al., 2004) |
|  | Livestock with dull coat (%) | D = 0 | (Huxley et al., 2004) |
|  | Livestock with thick hairy coat (%) | D = 15.8 | (Huxley et al., 2004) |
|  | Livestock with hock hair loss (%) | D = 78.6 | (Huxley et al., 2004) |
|  | Livestock with swollen hock (%) | D = 58.3 | (Huxley et al., 2004) |
|  | Livestock with ulcerated hock (%) | D = 5.3 | (Huxley et al., 2004) |
|  | Livestock with injury to inside hock (%) | D = 0 | (Huxley et al., 2004) |
|  | Livestock with injury to point of hock (%) | D = 41.7 | (Huxley et al., 2004) |
|  | Livestock with injury to hip bone (%) | D = 22.2 | (Huxley et al., 2004) |
|  | Livestock with injury to tail bone (%) | D = 45.0 | (Huxley et al., 2004) |
|  | Livestock with injury to stifle (%) | D = 7.1 | (Huxley et al., 2004) |
|  | Livestock with injury to knees (%) | D = 50 | (Huxley et al., 2004) |
|  | Livestock with injury to back and ribs (%) | D = 20 | (Huxley et al., 2004) |
|  | Livestock with injury to neck (%) | D = 50 | (Huxley et al., 2004) |
|  | Livestock with other injury (%) | D = 10 | (Huxley et al., 2004) |
|  | Maximum flight distance (m) | D = 2 | (Huxley et al., 2004) |
|  | Average flight distance (m) | D = 1.1 | (Huxley et al., 2004) |
|  | Livestock cows idling (%) | D = 3.5 | (Huxley et al., 2004) |
| Financial | Net farm income (£/ha) | D = £200 (1999)  Lowland B&S = -£81 (1999)  Less favoured areas C&S = £72 (1999) | (Fowler et al., 2001) |
|  | Management and investment income (£/ha) | D = £91 (1999)  Lowland B&S = -£182 (1999)  Less favoured areas cattle and sheep = £2 (1999) | (Fowler et al., 2001) |
|  | cash income (£/ha) | D = £245 (1999)  B&S = £222 (Lowland) (1999)  C&S = £156 (Less favoured areas) (1999) | (Fowler et al., 2001) |
|  | Gross margin before forage costs (£/head) | D = £1159 (1999)  B = £280 (lowland) (1999)  B (suckler) = £303 (less favoured areas) (1999)  B (finishing) = £239 (1999)  S = £54.4 (lowland) (1999)  S = £533.55 (upland) (1999) | (Fowler et al., 2001) |
|  | Farm profit (£/head) | D = £983 (2003) | (Häring, 2003) |
| Productivity | Milk yield (l/year/cow) | D = 5392  D = 5924 | (Fowler et al., 2001; Roderick et al., 1996) |
|  | Mean mortality (%) | S = 5.72 (lambs) | (Roderick et al., 1996) |

References

Chylinski, C., Athanasiadou, S., Thueer, S., Grovermann, C., Moakes, S., Hoste, H., Petkevicius, S., Verwer, C., Verkaik, J., & Werne, S. (2023). Reducing anthelmintic inputs in organic farming: Are small ruminant farmers integrating alternative strategies to control gastrointestinal nematodes? *VETERINARY PARASITOLOGY*, *315*. https://doi.org/10.1016/j.vetpar.2022.109864

Fowler, susan, Wynne-Jones, I., & Lampkin, N. (2001). *Organic Farm Incomes in England and Wales 1998/99*. Univesity of Aberystwyth.

Häring, A. M. (2003). Organic dairy farms in the EU: Production systems, economics and future development. *Livest. Prod. Sci.*, *80*(1–2), 89–97. https://doi.org/10.1016/S0301-6226(02)00308-1

Huxley, J. N., Burke, J., Roderick, S., Main, D. C. J., & Whay, H. R. (2004). Animal welfare assessment benchmarking as a tool for health and welfare planning in organic dairy herds. *Veterinary Record*, *155*(8), 237–239. https://doi.org/10.1136/vr.155.8.237

Roderick, S., Shart, N., & Hovi, M. (1996). Animal health and welfare research priorities. In *Organic Livestock Production* (pp. 30–50).
