# Supplementary material for: Outcomes of management practices on regenerative and organic cattle and sheep farms in the UK—a systematic literature review
Source: Org Agric. Author manuscript; Available in PMC 2026 Aug 15. (PMC7619368; doi:10.1007/s13165-026-00559-3)
Supplement: Supplementary file 2 [file EMS216180-supplement-Appendix_2.docx]

Appendix 1. Practices used on regenerative and organic beef (B), sheep (S) and dairy (D) farms in the UK. Empty boxes indicate that no information was available. GLU = Grazing Livestock Units.

| Category | Practice | Regenerative | Reference | Organic | Reference |
| --- | --- | --- | --- | --- | --- |
| Grazing management | Rotational grazing | B = 2  D = 0  S = 1 | (Jordon et al., 2023; Trickett & Warner, 2022) | B = 3  D = 4  S = 3 | (Ellis et al., 2011; Frost et al., 2009; Jackson et al., 2017; Maggs et al., 2008; Shell & Younie, 2001; Vine & Bateman, 1981; Weller & Bowling, 2000) |
|  | Continuous grazing/ set stocking |  |  | B = 0  D = 1  S = 0 | (Weller & Bowling, 2000) |
|  | Mixed-species grazing |  |  | B = 4  D = 2  S = 6 | (Fraser et al., 2013; Frost et al., 2009; Maggs et al., 2008; Roderick et al., 1996, 1999; Shell & Younie, 2001) |
|  | Integrated crop-livestock (on temporary grass leys or cover crops) | B = 2  D = 0  S = 1 | (Jordon et al., 2023; Trickett & Warner, 2022) |  |  |
| Stocking density | Low stocking density  (<=2 GLU per ha) |  |  | B = 5  D = 4  S = 3 | (Adamson, 2002; Fraser et al., 2013; Frost et al., 2009; Häring, 2003; Hovi & Kossaibati, 2002; Maggs et al., 2008; Shell & Younie, 2001) |
| Pasture composition | Perennial ryegrass |  |  | B = 0  D = 1  S = 0 | (Weller & Bowling, 2004) |
|  | Grass – clover mixtures | B = 1  D = 0  S = 0 | (Trickett & Warner, 2022) | B = 2  D = 5  S = 2 | (Frost et al., 2009; Vine & Bateman, 1981; Weller & Bowling, 2000, 2004) |
|  | Herbal leys | B = 1  D = 0  S = 1 | (Jordon et al., 2023) |  |  |
| Feed | Concentrates |  |  | B = 7  D = 8  S = 3 | (Adamson, 2002; Fowler et al., 2001; Frost et al., 2009; Keatinge, 2001; Langford et al., 2011; F. Langford et al., 2009; Roderick et al., 1999; Shell & Younie, 2001; Vine & Bateman, 1981; Weller & Bowling, 2000, 2004) |
|  | Fodder |  |  | B = 1  D = 1  S = 0 | (Hovi & Kossaibati, 2002) |
|  | Conserved forage (hay and silage) |  |  | B = 5  D = 6  S = 5 | (Adamson, 2002; Frost et al., 2009; Hovi & Kossaibati, 2002; Keatinge, 2001; Langford et al., 2011; Langford et al., 2009; Shell & Younie, 2001; Vine & Bateman, 1981; Weller & Bowling, 2000) |
|  | Forage and roughage |  |  | B = 1  D = 3  S = 1 | (Ellis et al., 2007; Hovi & Kossaibati, 2002; Shell & Younie, 2001; Weller & Bowling, 2000; Weller & Cooper, 1996a) |
|  | Straw |  |  | B = 1  D = 1  S = 1 | (Vine & Bateman, 1981) |
|  | Organic feed |  |  | B = 0  D = 1  S = 0 | (Rutherford et al., 2009) |
| Minerals and supplements | Seaweed meal |  |  | B = 1  D = 2  S = 0 | (Shell & Younie, 2001; Weller & Bowling, 2000, 2004) |
|  | Mineral Supplements |  |  | B = 2  D = 2  S = 0 | (Frost et al., 2009; Shell & Younie, 2001; Weller & Bowling, 2000, 2004) |
|  | Protein supplements |  |  | B = 0  D = 0  S = 1 | (Chylinski et al., 2023) |
| Housing | Part-housed |  |  | B = 5  D = 12  S = 0 | (Ellis et al., 2007, 2011; Frost et al., 2009; Haskell et al., 2009; Huxley et al., 2003; Jackson et al., 2017; Keatinge, 2001; Langford et al., 2011; Langford et al., 2009; Roderick et al., 1996, 1999; Rutherford et al., 2008; Shell & Younie, 2001; Vine & Bateman, 1981; Weller & Bowling, 2000, 2004; Weller & Cooper, 1996a) |
|  | Bedding in indoor housing |  |  | B = 1  D = 1  S = 1 | (Hovi & Kossaibati, 2002; Shell & Younie, 2001) |
|  | Outwintered |  |  | B = 2  D = 0  S = 2 | (Roderick et al., 1996, 1999; Shell & Younie, 2001) |
| Medication | Preventative health strategies |  |  | B = 0  D = 0  S = 1 | (Shell & Younie, 2001) |
|  | Therapeutic treatment with alternative remedies (e.g. herbal treatments, homeopathy) |  |  | B = 0  D = 10  S = 2 | (Chylinski et al., 2023; Hovi & Roderick, 2000; Langford et al., 2009; Roderick et al., 1996, 1999; Rutherford et al., 2008; Weller & Bowling, 2000; Weller & Cooper, 1996a, 1996b; Weller & Davies, 1998) |
|  | Foot baths |  |  | B = 1  D = 2  S = 2 | (Langford et al., 2009; Roderick et al., 1996; Weller & Davies, 1998) |
|  | Therapeutic treatment with pharmaceutical medication e.g. antibiotics |  |  | B = 2  D = 10  S = 3 | (Beynon et al., 2015; Chylinski et al., 2023; Ellis et al., 2011; Frost et al., 2009; Hovi & Roderick, 2000; Jackson et al., 2017; Langford et al., 2009; Maggs et al., 2008; Roderick et al., 1996, 1999; Rutherford et al., 2008; Shell & Younie, 2001; Weller & Cooper, 1996a) |
|  | Vaccinations e.g. for lungworm, Pasteurella etc. |  |  | B = 1  D = 4  S = 3 | (Ellis et al., 2011; Frost et al., 2009; Jackson et al., 2017; Roderick et al., 1996, 1999; Shell & Younie, 2001) |
| Reproduction | All year-round calving/lambing |  |  | B = 0  D = 3  S = 0 | (Ellis et al., 2011; Weller & Bowling, 2000; Weller & Cooper, 1996b) |
|  | Summer calving/lambing |  |  | B = 1  D = 1  S = 0 | (Ellis et al., 2011; Shell & Younie, 2001) |
|  | Spring calving/lambing |  |  | B = 4  D = 1  S = 3 | (Adamson, 2002; Frost et al., 2009; Jackson et al., 2017; Keatinge, 2001; Roderick et al., 1999; Shell & Younie, 2001) |
|  | Autumn calving/lambing |  |  | B = 0  D = 1  S = 0 | (Jackson et al., 2017) |
|  | Winter weaning |  |  | B = 2  D = 0  S = 0 | (Frost et al., 2009; Shell & Younie, 2001) |
|  | Summer weaning |  |  | B = 0  D = 0  S = 1 | (Frost et al., 2009) |
|  | Ealy weaning <6 weeks |  |  | B = 0  D = 1  S = 0 | (Roderick et al., 1996) |
|  | Late weaning >10 weeks |  |  | B = 1  D = 1  S = 0 | (Keatinge, 2001; Roderick et al., 1996) |
|  | Natural milk for young |  |  | B = 1  D = 1  S = 0 | (Hovi & Kossaibati, 2002) |
|  | Artificial insemination |  |  | B = 2  D = 3  S = 0 | (Roderick et al., 1996, 1996; Weller & Bowling, 2000) |
|  | Natural service |  |  | B = 2  D = 2  S = 0 | (Roderick et al., 1996, 1999) |
|  | Birthing indoors |  |  | B = 0  D = 0  S = 3 | (Frost et al., 2009; Roderick et al., 1996, 1999) |
|  | Birthing outdoors |  |  | B = 1  D = 0  S = 2 | (Roderick et al., 1996, 1999; Shell & Younie, 2001) |
|  | Single and twin rearing livestock graze separately |  |  | B = 0  D = 0  S = 2 | (Frost et al., 2009; Keatinge, 2001) |
| Inputs – feed and bedding | Bought in | S = 1  B = 1  D = 0 | (Jordon et al., 2023) | B = 0  D = 1  S = 2 | (Frost et al., 2009; Shell & Younie, 2001; Vine & Bateman, 1981) |
|  | Produced on farm | B = 1  D = 0  S = 1 | (Jordon et al., 2023) | B = 1  D = 1  S = 1 | (Frost et al., 2009; Shell & Younie, 2001; Weller & Cooper, 1996a) |
|  | No tillage | B = 1  D = 0  S = 0 | (Trickett & Warner, 2022) |  |  |
| Fertiliser | Mineral fertiliser |  |  |  |  |
|  | Organic fertiliser |  |  | B = 1  D = 1  S = 1 | (Vine & Bateman, 1981) |
| Grooming | Foot trimming |  |  | B = 0  D = 2  S = 0 | (Haskell et al., 2009; Roderick et al., 1996) |
| Milking | Standard milking frequency – twice per day |  |  | B = N/A  D = 2  S = N/A | (Haskell et al., 2009; Rutherford et al., 2008) |
| Biodiversity | Integrate trees on farm | B = 1  D = 0  S = 1 | (Jordon et al., 2023) |  |  |
|  | Hedgerows | B = 1  D = 0  S = 1 | (Jordon et al., 2023) |  |  |

References

Adamson. (2002). *Defra Final project report: Organic beef and sheep production in the uplands*. https://orgprints.org/id/eprint/6594/

Beynon, S., Wainwright, W., & Christie, M. (2015). The application of an ecosystem services framework to estimate the economic value of dung beetles to the UK cattle industry. *Ecological Entomology*, *40*, 124–135. (WOS:000360220600012). https://doi.org/10.1111/een.12240

Chylinski, C., Athanasiadou, S., Thueer, S., Grovermann, C., Moakes, S., Hoste, H., Petkevicius, S., Verwer, C., Verkaik, J., & Werne, S. (2023). Reducing anthelmintic inputs in organic farming: Are small ruminant farmers integrating alternative strategies to control gastrointestinal nematodes? *Veterinary Parasitology*, *315*. (WOS:000976675100001). https://doi.org/10.1016/j.vetpar.2022.109864

Ellis, K. A., Innocent, G., Mihm, M., Cripps, P., McLean, W. G., Howard, C. V., & Grove White, D. (2007). *Dairy cow cleanliness and milk quality on organic and conventional farms in the UK*. *74*(3), 302–310. https://doi.org/10.1017/S002202990700249X

Ellis, K. A., Jackson, A., Bexiga, R., Matthews, J., McGoldrick, J., Gilleard, J., & Forbes, A. B. (2011). Use of diagnostic markers to monitor fasciolosis and gastrointestinal nematodes on an organic dairy farm. *Veterinary Record*, *169*(20), 524. Scopus. https://doi.org/10.1136/vr.d5021

Fowler, S., Wynne-Jones, I., & Lampkin, N. (2001). *Organic Farm Incomes in England and Wales 1998/99*. Univesity of Aberystwyth.

Fraser, M., Vale, J., & Firbank, L. (2013). Effect on Habitat Diversity of Organic Conversion Within the Less Favored Areas of England and Wales. *Agroecology and Sustainable Food Systems*, *38*(2), 243–261. (WOS:000327583900006). https://doi.org/10.1080/21683565.2013.849320

Frost, D., Morgan, M., & Moakes, S. (2009). *A farmer’s guide to Organic upland beef and sheep production (Organic farming technical guide)*. Prifysgol Aberystwyth | Aberystwyth University.

Häring, A. (2003). Organic dairy farms in the EU: Production systems, economics and future development. *LIVESTOCK PRODUCTION SCIENCE*, *80*(1–2), 89–97. (WOS:000182149600009). https://doi.org/10.1016/S0301-6226(02)00308-1

Haskell, M., Langford, F., Jack, M., Sherwood, L., Lawrence, A., & Rutherford, K. (2009). The effect of organic status and management practices on somatic cell counts on UK dairy farms. *Journal of Dairy Science*, *92*(8), 3775–3780. (WOS:000268136400024). https://doi.org/10.3168/jds.2009-2105

Hovi, M., & Kossaibati, M. (2002). The impact of organic standards on cattle health and welfare—A survey of veterinarians, advisors and inspectors. *Cattle Practice*, *10*(3), 183–189.

Hovi, M., & Roderick, S. (2000). *Mastitis and mastitis control strategies in organic milk*. *8*(3), 259–264.

Huxley, J., Burke, J., Roderick, S., Main, D., & Whay, H. (2003). Herd health and welfare benchmarking on organic dairy farms in South-West England. *Cattle Practice*, *11*, 331–333. (WOS:000186749400019).

Jackson, A., Ellis, K. A., McGoldrick, J., Jonsson, N. N., Stear, M. J., & Forbes, A. B. (2017). Targeted anthelmintic treatment of parasitic gastroenteritis in first grazing season dairy calves using daily live weight gain as an indicator. *Veterinary Parasitology*, *244*, 85–90. Scopus. https://doi.org/10.1016/j.vetpar.2017.07.023

Jordon, M., Winter, D., & Petrokofsky, G. (2023). Advantages, disadvantages, and reasons for non-adoption of rotational grazing, herbal leys, trees on farms and ley-arable rotations on English livestock farms. *AGROECOLOGY AND SUSTAINABLE FOOD SYSTEMS*, *47*(3), 330–354. (WOS:000889091800001). https://doi.org/10.1080/21683565.2022.2146253

Keatinge, R. (2001). *Organic beef and sheep production (OF0147)*.

Langford, F. M., Rutherford, K. M., Jack, M. C., Sherwood, L., Lawrence, A. B., & Haskell, M. J. (2009). A comparison of management practices, farmer-perceived disease incidence and winter housing on organic and non-organic dairy farms in the UK. *Journal of Dairy Research*, *76*(1), 6–14. https://doi.org/10.1017/S0022029908003622

Langford, F. M., Rutherford, K., Sherwood, L., Jack, M., Lawrence, A., & Haskell, M. (2011). Behavior of cows during and after peak feeding time on organic and conventional dairy farms in the United Kingdom. *Journal of Dairy Science*, *94*(2), 746–753. (WOS:000286407600019). https://doi.org/10.3168/jds.2010-3309

Maggs, L., Athanasiadou, S., Sherwood, L., & Haskell, M. (2008). Levels of parasitism on organic and non-organic dairy farms in Scotland. *Veterinary Record*, *162*(11), 345–346. (WOS:000254594200014). https://doi.org/10.1136/vr.162.11.345

Roderick, S., Hovi, M., & Short, N. (1999). Animal health and welfare issues on organic livestock farms in the UK: results of a producer survey. *BSAP Occasional Publication*, *23*, 109–112.

Roderick, S., Shart, N., & Hovi, M. (1996). Animal health and welfare research priorities. In *Organic Livestock Production* (pp. 30–50).

Rutherford, Langford, F., Jack, M., Sherwood, L., Lawrence, A., & Haskell, M. (2008). Hock injury prevalence and associated risk factors on organic and nonorganic dairy farms in the United Kingdom. *Journal of Dairy Science*, *91*(6), 2265–2274. (WOS:000255945800012). https://doi.org/10.3168/jds.2007-0847

Rutherford, Langford, F., Jack, M., Sherwood, L., Lawrence, A., & Haskell, M. (2009). Lameness prevalence and risk factors in organic and non-organic dairy herds in the United Kingdom. *Veterinary Journal*, *180*(1), 95–105. (WOS:000264498000014). https://doi.org/10.1016/j.tvjl.2008.03.015

Samuel, A., & Dines, L. (Eds.). (2023). 21—Grazing management. In *Lockhart and Wiseman’ s Crop Husbandry Including Grassland (Tenth Edition)* (pp. 585–603). Woodhead Publishing. https://doi.org/10.1016/B978-0-323-85702-4.00006-6

Shell, D., & Younie, D. (2001). Organic beef production: Two case studies of rearing/finishing enterprises. In *Organic Livestock Farming*.

Takahashi, T., Harris, P., Blackwell, M., Cardenas, L., Collins, A., Dungait, J., Hawkins, J., Misselbrook, T., McAuliffe, G., McFadzean, J., Murray, P., Orr, R., Rivero, M., Wu, L., & Lee, M. (2018). Roles of instrumented farm-scale trials in trade-off assessments of pasture-based ruminant production systems. *Animal*, *12*(8), 1766–1776. (WOS:000439556700024). https://doi.org/10.1017/S1751731118000502

Trickett, T., & Warner, D. (2022). Earthworm Abundance Increased by Mob-Grazing Zero-Tilled Arable Land in South-East England. *Earth*, *3*(3), 895–906. (WOS:001109143000001). https://doi.org/10.3390/earth3030052

Vine, A., & Bateman, D. (1981). *Organic farming systems in England and Wales: Practices, performance and implications.* University College of Wales Aberystwyth.

Weller, R. F., & Bowling, P. J. (2000). Health status of dairy herds in organic farming. *Veterinary Record*, *146*, 80–81. https://doi.org/10.1136/vr.146.3.80

Weller, R. F., & Bowling, P. J. (2004). The Performance and Nutrient Use Efficiency of Two Contrasting Systems of Organic Milk Production. *Biological Agriculture & Horticulture*, *22*(3), 261–270. https://doi.org/10.1080/01448765.2004.9755289

Weller, R. F., & Cooper, A. (1996a). Animal health. In *Conversion to organic milk production* (4th ed.).

Weller, R. F., & Cooper, A. (1996b). Health status of dairy herds converting from conventional to organic dairy farming. *The Veterinary Record*, *139*(6), 141–142. https://doi.org/10.1136/vr.139.6.141

Weller, R. F., & Davies, D. W. (1998). Somatic cell counts and incidence of clinical mastitis in organic milk production. *The Veterinary Record*, *143*(13), 365–366. https://doi.org/10.1136/vr.143.13.365
