# Supplementary material for: Outcomes of management practices on regenerative and organic cattle and sheep farms in the UK—a systematic literature review
Source: Org Agric. Author manuscript; Available in PMC 2026 Aug 15. (PMC7619368; doi:10.1007/s13165-026-00559-3)
Supplement: Supplementary file 3 [file EMS216180-supplement-Appendix_3.docx]

Appendix 3. Opinions of the impacts of regenerative and organic (B), sheep (S) and dairy (D) system on animal health, animal welfare, biodiversity, economics and productivity.

| Farming system | Positive impact | No impact or mixed opinions | Negative impact |
| --- | --- | --- | --- |
| Organic | Health and welfare | | |
|  | Increased positive attitudes to AMR mitigation^D^(Borelli et al., 2023) | Use of health and welfare records^BD^  (Hovi & Kossaibati, 2002) | Footrot^S^, mastitis^DS^, fly strike^S^, fluke^S^ and worms^S^ (Roderick et al., 1999) |
|  | Clean or mixed grazing^S^ (Roderick et al., 1999) | Prohibition of artificial reproduction^BD^  (Hovi & Kossaibati, 2002) | Deficiencies in trace elements ^S^ (Roderick et al., 1999) |
|  | Ample dry bedding^BD^ (Hovi & Kossaibati, 2002) |  | Prohibition to use conventional medicine^BD^  (Hovi & Kossaibati, 2002) |
|  | Low stocking density^BD^ (Hovi & Kossaibati, 2002) |  | Restriction on the number of individual medical treatments^BD^  (Hovi & Kossaibati, 2002) |
|  | Solid floor area not slatted or grid, breeds selected to avoid health problems construction^BD^ (Hovi & Kossaibati, 2002) |  | Prohibition of organophosphates^BD^  (Hovi & Kossaibati, 2002) |
|  | At least 60% dry matter consisting of roughage, fodder or silage^BD^ (Hovi & Kossaibati, 2002) |  |  |
|  | High quality feed^BD^ (Hovi & Kossaibati, 2002) |  |  |
|  | Regular exercise^BD^ (Hovi & Kossaibati, 2002) |  |  |
|  | Access to pasture^BD^ (Hovi & Kossaibati, 2002) |  |  |
|  | Natural milk for feeding young^BD^ (Hovi & Kossaibati, 2002) |  |  |
|  | Forbidding calves in individual pens^BD^ (Hovi & Kossaibati, 2002) |  |  |
|  | Economics | | |
|  |  | Increase in income ^BDS^ (Tranter et al., 2007) |  |
| Regenerative | Productivity | | |
|  | Rotational grazing ^BS^ (Jordon et al., 2023) |  |  |
|  | Productivity and animal health | | |
|  | Herbal leys ^BS^ (Jordon et al., 2023) |  |  |
|  | Welfare, finance, biodiversity | | |
|  | Trees on pasture^BS^ (Jordon et al., 2023) |  |  |

References

Borelli, E., Ellis, K., Pamphilis, N., Tomlinson, M., & Hotchkiss, E. (2023). Factors influencing Scottish dairy farmers’ antimicrobial usage, knowledge and attitude towards antimicrobial resistance. *Preventive Veterinary Medicine*, *221*. (WOS:001113350400001). https://doi.org/10.1016/j.prevetmed.2023.106073

Hovi, M., & Kossaibati, M. (2002). The impact of organic standards on cattle health and welfare—A survey of veterinarians, advisors and inspectors. *Cattle Practice*, *10*(3), 183–189.

Jordon, M., Winter, D., & Petrokofsky, G. (2023). Advantages, disadvantages, and reasons for non-adoption of rotational grazing, herbal leys, trees on farms and ley-arable rotations on English livestock farms. *AGROECOLOGY AND SUSTAINABLE FOOD SYSTEMS*, *47*(3), 330–354. (WOS:000889091800001). https://doi.org/10.1080/21683565.2022.2146253

Roderick, S., Hovi, M., & Short, N. (1999). Animal health and welfare issues on organic livestock farms in the UK: results of a producer survey. *BSAP Occasional Publication*, *23*, 109–112.

Tranter, R., Holt, G., & Grey, P. (2007). Budgetary implications of, and motives for, converting to organic farming: Case study farm business evidence from Great Britain. *Biological Agriculture & Horticulture*, *25*(2), 133–151. (WOS:000252674000004). https://doi.org/10.1080/01448765.2007.9755042
